# Clinicians should be aware of their responsibilities as role models: a case report on the impact of poor role modeling

**DOI:** 10.3402/meo.v19.23479

**Published:** 2014-02-04

**Authors:** Lukas P. Mileder, Albrecht Schmidt, Hans P. Dimai

**Affiliations:** 1 Clinical Skills Center, Medical University of Graz, Graz, Austria; 2 Division of Cardiology, Department of Internal Medicine, Medical University of Graz, Graz, Austria; 3 Division of Endocrinology and Metabolism, Department of Internal Medicine, Medical University of Graz, Graz, Austria; 4 Vice-Rectorate for Teaching and Studies, Medical University of Graz, Graz, Austria

**Keywords:** medical education, role modeling, professional behavior, faculty development

## Abstract

**Background:**

Role modeling is an important and valuable educational method. It is predominant throughout (under-)graduate medical education, and attributes of exemplary medical role models are manifold.

**Aim:**

This article describes the impact of poor role modeling on medical students’ professional and personal development on the basis of a singular incident at an associated teaching hospital. In addition, scientific literature studying the effect of and the reasons behind poor role modeling in undergraduate and graduate medical education is analyzed and discussed.

**Results:**

To maximize the educational potential of clinical role modeling, medical schools have to consider strategies both on the individual as well as on the institutional level. Several suggestions are offered on both levels.

**Discussion/conclusion:**

Based on a case report of significantly poor role modeling, this article outlines strategies through which academic medical institutions may maximize the educational potential of role modeling and lastingly enhance teaching proficiency of clinical faculty.

## Background

Role modeling, or teaching by example, has been identified as an important educational method that medical students encounter throughout undergraduate training (1). Role models are ‘people we can identify with, who have qualities we would like to have, and are in positions we would like to reach’ (2). Attributes of exemplary medical role models include spending a considerable amount of time teaching and conducting rounds, emphasizing the importance of the doctor–patient relationship while teaching, and integrating psychosocial aspects of medicine (3). The importance of role modeling is highlighted by the fact that 90% of students identify one or multiple role model(-s) during medical school (4).

## The impact of poor role modeling

At our medical school, all first-year students are obliged to participate in an 11-hour skills tutorial, featuring practical contents that are of importance for students during clinical electives and clerkships. A central course objective is to teach students the importance of doctor–patient communication and simple measures of patient safety (e.g., correct patient identification, compliance with skin disinfection instructions).

Months after having successfully completed the course, two students visited our skills laboratory for additional practical training. The attending student instructor recognized distinct insecurity regarding blood sample taking and venous cannulation, procedures which are trained in depth as part of the tutorial. During an informal conversation, the students disclosed that their insecurity was due to an event that happened on the first day of their first clinical elective, which took place at an internal medicine department of an associated non-university teaching hospital. Both students were assigned to an intern whom they followed during his morning routine, consisting of taking blood samples and placing intravenous lines. During these patient encounters, the students recognized major deficiencies in medical practice and distinct interpersonal misbehavior on behalf of the intern. He never introduced himself to patients, just casually offered words of welcome while already preparing the equipment, and regularly used the phrase ‘we need some blood for tests’ instead of explaining the need for the procedure. When questions arose, the intern referred to his workload and asked patients to consult ‘some students or nurses’. During at least four patient encounters, the intern neither changed his examination gloves nor cleaned the equipment tray once. Students reported that skin disinfection consisted of ‘touching the planned puncture site with the disinfection swab for mere seconds’. During lunch break, the students asked the intern about the differences between his way of practicing and what they had learned. Allegedly, the intern told them with a sarcastic smile, ‘That's good in the skills lab. This, however, is the real world with a different set of practices and rules’.

During the subsequent 3 weeks, the students observed many other clinicians during their daily practice. They described most of these encounters as positive, but still this first experience had left its mark. Both students acknowledged that they actively avoided practical training opportunities. The confusion about whose example to follow made them feel desperate and helpless. When asked why they had not contacted any other clinical teacher, fear of negative consequences for themselves and shame about their inexperience were named as reasons.

It can be assumed that this event is no singular case and, therefore, medical schools have to identify strategies to prevent such happenings. Jackson et al. (5) reported that despite the presence of students, clinical staff are not always willing or capable of performing aseptic practice correctly. In some instances, students were instructed by clinicians to deliberately perform bad practice, causing even more concern. Patient harm as a result of medical care is common, with nosocomial infections and procedure-related consequences being among the most frequent causes (6). Active failures (errors and deviations from policies) and individual factors (e.g., inexperience, stress, personality) have been identified as the main reasons for patient safety incidents (7).

A survey among medical students reported that 61% had witnessed unethical behavior by medical team members at least once (8). These students were much more likely to have acted unethically themselves. Sixty-two percent of the students agreed that some of their ethical principles had been eroded or lost since starting medical school, and 38% stated displeasure with their personal ethical development.

## Discussion

Most clinical staff members are aware of their responsibilities as teachers and role models, and hold themselves to high standards. The aforementioned examples of poor role modeling may be due to clinicians’ workload, inexperience, lack of medical knowledge or little motivation to teach. Inappropriate conduct may also have its root in the fact that both students and residents often do not accurately judge their own behavior as (un-)professional (9, 10). Regardless of reasons, poor role modeling can cause inappropriate or even unethical behavior (8, 11–13). On the other hand, a recent study suggested that observation of so-called anti-models may represent a valuable experience for students, as it could serve as an effective mechanism of social learning (14). Still, as medical training is not ‘just about the acquisition of new knowledge and skills, but about the acquisition of a physician identity and character’ (11), this ‘hidden curriculum’ of at times improper conduct and practice needs to be countered by good role modeling. So how is this to be achieved?

Role modeling starts with individuals and reaches to the administration level of academic institutions. Therefore, in order to actively enhance the quality of clinical teaching and role modeling, strategies both on the individual and organizational level have to be considered. Suggestions are summarized in Table 1.

**Table 1:** Strategies for improvement of clinical role modeling

Raise awareness among clinical faculty on teaching responsibilities
Implementation of development programs on active teaching, delivering feedback, professional behavior, and ethics both in undergraduate and graduate curricula
Training of clinical teachers in self-reflective practice
Provision of institutional support (administrative, financial)
Allocation of sufficient teaching time and focus on small-group teaching
Formal institutional recognition of dedicated clinical teachers
Standardization of teaching contents and communication of clinical learning objectives
Thorough assessment of organizational ‘framework’ and of explicit as well as implicit teaching and learning

First and foremost, faculty members have to consciously realize that all of their interactions, personal opinions, and attitudes affect the students who follow and observe them (1), as role modeling occurs in any situation in which a student observes clinical staff. Didactic training, peer-mentoring programs for clinical teachers, and structured supervision by experienced educators may help to raise awareness among healthcare professionals about the importance of role modeling.

Clinical teachers have to intellectually challenge students while always maintaining a supportive attitude (15). Providing deliberate feedback on students’ performance is another vital component of their learning experience (16). However, engaging students in active learning and delivering feedback in a constructive and sensitive manner requires both time and training. Therefore, medical schools should: 1) implement mandatory development programs for all staff members engaged in student education and 2) allow for sufficient feedback time in courses and clinical placements. It has been suggested to use video analyses for didactic training to provide clinical teachers a mechanism for self-discovery and improvement (17). Professional behavior and ethics should be dealt with during these trainings comprehensively (2). Such programs lead to significantly improved teaching quality among clinical staff (18). As today's medical students are the clinical teachers of tomorrow, educational interventions aiming at developing students’ teaching skills should also be integrated in undergraduate curricula (19).

Self-reflection may be the primary method by which clinical teachers develop and increase their educational proficiency (13). Reflective practice has been identified as an effective instrument of personal and professional development; organized activities such as Balint groups may enhance physicians’ personal awareness, which can lead to behavioral changes and help improving individual satisfaction, patient care, and clinical teaching quality (20).

Even the most skilled and enthusiastic teachers have to rely on institutional support in order to perform their craft at the highest level. This includes manifold aspects such as providing administrative help, financial resources, time for preparation and teaching itself, and, last but not least, institutional appreciation of those aiming to be exemplary. Availability of sufficient teaching time is highly critical to effective role modeling, as it requires dialogue, reflection, and debriefing to render lessons learnt through role modeling perceptible to the learner (21).

Standardization of teaching content may constitute another approach to improve clinical teaching. Academic institutions should draw up and communicate clinical learning objectives and ensure adherence to these standards. In addition, medical schools should provide healthcare teams with information on the experience of each student prior to clinical placements (15).

Finally, it has to be remembered that medical education encompasses a formal, an informal, and a hidden curriculum (11, 12). Role modeling takes place in all three areas. Medical schools have to take education behind formal curricula and course objectives seriously, as negative role modeling may have an especially serious impact within the random, unplanned, and highly interactive informal curriculum (21). Institutional culture such as a lack of cohesiveness within healthcare teams, pronounced hierarchies, inadequate patient care, and organizational structures tolerating overwork or favoring research over teaching may also prevent positive role modeling (21). Therefore, academic institutions should thoroughly assess their organizational framework and deliberately investigate not only official curricula but also the implicit teaching and learning that takes place in hallways, locker rooms and cafeterias during lunch breaks and night shifts. While doing so, administrators should keep the following suggestion in mind, ‘Educators need to focus less on what is taught in medical school and more on what is being learned’ (12).

## Conclusion

Every medical teacher should strive to be a positive role model, as educating future physicians is not only a privilege but also an obligation. Academic medical institutions have to realize that they are represented by every member of their teaching staff, be it in the classroom or at the patients’ bedside, and that clinical teachers need skills, time, and care in order to display exemplary behavior (17). Therefore, thorough self-evaluation, as well as the installation and protection of organizational structures aiming at enhancing each teaching individual's motivation and proficiency, have to be every medical school's primary objectives.
